# Percutaneous Left Atrial Appendage Closure in Patients With Cardioembolic Breakthrough Stroke: An International Observational Study

**DOI:** 10.1111/ene.70365

**Published:** 2025-09-23

**Authors:** Roberto Galea, Gavino Casu, Tommaso Bini, Angelo Laconi, Pier Luigi Merella, Konstantina Chalkou, Alberto Preda, Marco Cerciello, Giuseppe D'Angelo, Paolo della Bella, Patrizio Mazzone, Elias Auer, Antanas Gasys, David Julian Seiffge, Urs Fischer, Lorenz Räber

**Affiliations:** ^1^ Department of Cardiology, Bern University Hospital University of Bern Bern Switzerland; ^2^ Department of Cardiology Hospital Centre of Biel Biel Switzerland; ^3^ Clinical and Interventional Cardiology University of Sassari Sassari Italy; ^4^ Graduate School for Health Sciences University of Bern Bern Switzerland; ^5^ Department of Clinical Research University of Bern Bern Switzerland; ^6^ Electrophysiology Unit, De Gasperis Cardio Center Niguarda Hospital Milan Italy; ^7^ Department of Cardiac Electrophysiology and Arrhythmology IRCCS San Raffaele University Hospital Milan Italy; ^8^ Department of Neurology, Bern University Hospital University of Bern Bern Switzerland

**Keywords:** atrial fibrillation, breakthrough stroke, left atrial appendage closure, oral anticoagulation, stroke

## Abstract

**Background:**

Patients with non‐valvular atrial fibrillation (AF) who experience an ischemic stroke despite oral anticoagulation (OAC) are at particularly high risk of recurrence, with a reported annualized ischemic stroke rate of 5.3%–8.9%. The optimal strategy for secondary prevention in these patients remains unknown.

**Methods:**

We reviewed all percutaneous left atrial appendage closures (LAAC) attempted in AF patients experiencing an ischemic stroke under OAC and who were prospectively collected in four European centers. All index strokes were categorized by an experienced neurologist to exclude patients with non‐cardioembolic etiology or insufficient OAC. The primary endpoint was a recurrent ischemic stroke at 2 years after the procedure. Secondary endpoints included procedure‐related complications and 2‐year death.

**Results:**

Of 2234 patients submitted to LAAC procedure, 95 had a cardioembolic breakthrough stroke. LAAC procedures were performed at a mean of 4 months after the breakthrough stroke. The main antithrombotic therapy at discharge (83%) and at the latest follow‐up (79%) consisted of OAC. At the median follow‐up of 713 days, the primary endpoint occurred in 4 patients (4%). Procedure‐related complications were rare (1%) whereas death occurred in 5% of patients.

**Conclusion:**

LAAC procedures were safe and feasible in patients with cardioembolic breakthrough stroke. Recurrent stroke rates were lower than those reported in previous studies with OAC continuation after breakthrough stroke, suggesting a potential additive protection by LAAC on top of OAC. Results from ongoing randomized trials are required to validate our findings.

## Graphical abstract

Main Results of the Study.

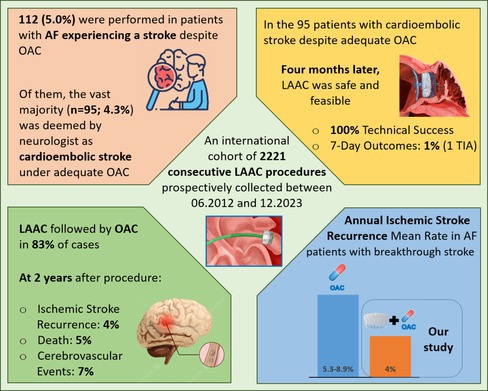

**Abbreviations**
AF: Atrial fibrillation
BARC: Bleeding Academic Research Consortium
CHA_2_DS_2_Vasc: congestive heart failure, hypertension, age ≥ 75, diabetes mellitus, prior stroke or transient ischemic attack, vascular disease, age 65–74, female
DOAC: direct oral anticoagulation
DRT: device related thrombus
LAA: left atrial appendage
LAAC: left atrial appendage closure
OAC: oral anticoagulation
PDL: peri‐device leak
TEE: transesophageal echocardiography
VKA: vitamin K antagonists

## 1 Introduction

Atrial fibrillation (AF) is the most common arrhythmia and a major cause of ischemic stroke [1]. Oral anticoagulation (OAC) is the most effective prophylaxis for stroke in AF [2]. However, its efficacy is not absolute, as in the large direct OAC (DOAC) approval trials, approximately 2% of AF patients on DOAC experienced a breakthrough stroke, defined as an ischemic stroke occurring despite optimal OAC [2]. Secondary prevention in such patients remains challenging due to an incomplete understanding of the underlying mechanisms [3]. These may include cardioembolic causes (e.g., cardioembolic breakthrough stroke), non‐cardioembolic pathways (e.g., lacunar stroke, large artery diseases, vasculitis, etc.), or insufficient OAC [4]. As changing the type of DOAC has not resulted in improved outcomes [5], the current ESC guidelines for AF recommend not changing the therapy (class III) [6]. Furthermore, breakthrough stroke patients are at a particularly high stroke recurrence risk of up to 9% per year, highlighting the unmet need for new therapeutic concepts [7].

Left atrial appendage (LAA) is the main source of thrombi in patients with AF [8, 9, 10]. A recent randomized clinical trial assessing the impact of surgical LAA closure (LAAC) on thromboembolic risk in AF patients undergoing cardiac surgery demonstrated that LAAC, in addition to OAC, further enhances protection against thromboembolic events compared to OAC alone [11]. However, evidence supporting percutaneous LAAC in patients with breakthrough stroke is limited. Several observational studies showed the feasibility and the safety of percutaneous LAAC in such patients with promising recurrence stroke rates [12, 13]. Yet, the inclusion of even patients experiencing non‐cardioembolic stroke or with reduced OAC compliance in the vast majority of studies limits the generalizability of their results, potentially underestimating the efficacy of LAAC in reducing stroke recurrence.

We therefore aim at reporting the long‐term clinical and imaging outcomes of a multicentre cohort of AF patients experiencing cardioembolic stroke despite optimal OAC and subsequently submitted to LAAC.

## 2 Methods

### 2.1 Study Design and Population

We conducted a multicentre observational study involving the following four European centers: Bern University Hospital (UH) (Switzerland), Sassari UH (Italy), San Raffaele Hospital (Italy), Niguarda Hospital (Italy). All consecutive LAAC attempted in the above centers between June 2012 and December 2023 and prospectively collected in local registries were considered for the current analysis. All participating centers identified and provided data on any AF patients submitted to LAAC following a cerebrovascular event despite OAC therapy. All index cerebrovascular events were adjudicated by an experienced neurologist according to current standards of care, and only those categorized as cardioembolic strokes despite adequate OAC were considered for this analysis [5]. The following patients were excluded:

1. patients with index ischemic stroke due to possible competing etiology (such as small vessel disease, large artery atherosclerosis, or other established pathologies [e.g., endocarditis] as the most likely stroke mechanism in line with the TOAST classification criteria, that is, ‘two or more mechanisms’) [14];
2. patients with medication issues, defined as at least one of the following criteria: self‐reported interruption of OAC for > 48 h; low anticoagulant activity on admission if available (e.g., International Normalized Ratio [INR] < 2 for Vitamin K Antagonists [VKA], etc.); inappropriately low DOAC dose or dosing frequency;
3. patients with hemorrhagic or undetermined stroke or transient ischemic attack (TIA) on OAC.

Data management and statistical analyses were performed by an independent biostatistician (KC) at the Clinical Trial Unit of Bern University Hospital. The study complies with the Declaration of Helsinki, and a local ethical approval was obtained at each of the participating centers.

### 2.2 LAAC Procedure and Follow‐Up

The data related to the index stroke, baseline and procedural patient characteristics, antithrombotic therapy during index stroke and after LAAC were collected. LAAC procedures were performed according to the local standard of care [15, 16, 17]. The LAAC device was chosen based on the LAA morphology and the preference of the operator [16, 18, 19]. The post‐implantation antithrombotic regimen was left to the discretion of the treating physician [20, 21, 22]. A transesophageal echocardiography (TEE) follow‐up was performed between 1 and 3 months after the intervention in order to exclude device‐related complications including thrombus (DRT) and peridevice leak (PDL). The clinical follow‐up was not standardized and was left up to the local centers. Patients were followed by phone, and, if possible, by means of an outpatient visit. Source documentation of all adverse events was collected, and all events were classified and adjudicated by trained cardiologists and neurologists in accordance with the events definitions provided by the coordinating center (Supporting Information). The 2‐year clinical follow‐up was reported.

### 2.3 Study Endpoints

The primary endpoint was recurrent ischemic stroke. Secondary endpoints included death, cerebrovascular events, procedure‐related complications (including death, cerebrovascular events, and major bleeding events that occurred within 7 days after the procedure), and DRT.

Three subanalyses were performed: first, cerebrovascular events of patients discharged with or without OAC were compared; second, cerebrovascular events of patients implanted with single versus double closure system devices were compared; third, the thromboembolic events (including stroke, TIA, or systemic embolisms) rate observed at 1 year was compared to that expected by the CHA_2_DS_2_‐VASc Score [23].

### 2.4 Statistical Analysis

Continuous variables are presented as mean ± standard deviation or as median with interquartile range, and categorical variables as frequencies and percentages. Cerebrovascular events of patients discharged with or without OAC or implanting single or double closure system devices were compared by using a chi‐square test. The expected thromboembolic event rate was calculated for each patient based on his/her CHA_2_DS_2_‐VASc score and the 1‐year thromboembolic event rate reported by a previous publication for AF patients with a similar CHA_2_DS_2_‐VASc score [23]. Expected and observed events were compared by using the Poisson Model. A *p* value < 0.05 was considered statistically relevant. IBM SPSS Statistics version 25 (IBM Corp) was used.

## 3 Results

A total of 2221 LAAC were reviewed (Figure S1). Of them, 112 (5.0%) were performed in patients experiencing a cerebrovascular event despite OAC. After neurological evaluation, the following patients were excluded: seven patients with a possible competing etiology, seven who were not under an optimal OAC regimen at the time of the index stroke, and three who had a non‐ischemic stroke (Figure S1). Therefore, 95 patients were identified as having a cardioembolic stroke despite optimal OAC and were included in our analysis.

The antithrombotic regimen at the time of the cerebrovascular accident was DOAC in 74 patients (78%) and VKA in 21 patients (22%) (Table 1). The index stroke generally preceded the LAAC procedure by 4 months (median: 120 days; IQL: 57–190 days).

**TABLE 1:** Index stroke and baseline characteristics.

	All patients (*N* = 95)
Days between index stroke and LAAC; median (IQR)	120.0 (57.0; 190.0)
**Oral anticoagulant during index stroke**	
DOAC, *n* (%)	74 (78%)
Apixaban, *n* (%)	24 (25%)
Rivaroxaban, *n* (%)	22 (23%)
Dabigatran, *n* (%)	21 (22%)
Edoxaban, *n* (%)	7 (7%)
VKA, *n* (%)	21 (22%)
Age (years), mean ± SD	74.1 ± 11.4
Female, *n* (%)	36 (38%)
BMI (kg/m^2^), mean ± SD	*N* = 91; 26.9 ± 3.9
Arterial hypertension, *n* (%)	73 (77%)
Diabetes mellitus, *n* (%)	27 (28%)
CHA_2_DS_2_‐VASc score, mean ± SD	5.4 ± 1.3
HAS‐BLED score, mean ± SD	3.0 ± 1.0
Paroxysmal AF, *n* (%)	44 (46%)
History of bleeding, *n* (%)	11 (12%)
Intracranial bleeding, *n* (%)	1 (1%)
Gastrointestinal bleeding, *n* (%)	7 (7%)
Others, *n* (%)	6 (6%)
History of chronic kidney disease^a^, *n* (%)	4 (4%)
History of hematological disease, *n* (%)	6 (6%)

Abbreviations: AF, atrial fibrillation; BMI, body mass index; DOAC, direct oral anticoagulant; IQR, interquartile range; LAAC, left atrial appendage closure; SD, standard deviation; VKA, vitamin K antagonist.

^a^ Defined as < 30 eGFR mL/min per 1.73 m^2^ (using the Modification of Diet in Renal Disease formula) and/or Creat > 200 mcmol/l and/or dialysis or history of kidney transplantation.

Baseline characteristics are summarized in Table 1. The majority of patients were male (*n* = 59, 62%) and had a mean age of 74.1 years. The mean CHA_2_DS_2_‐VASc score and the mean HAS‐BLED score were 5.4 ± 1.3 and 3.0 ± 1.0, respectively. More than one fourth of patients were diabetic (*n* = 27, 28%), and only in a minority of patients had a history of relevant bleeding was reported (*n* = 11, 12%).

Procedural characteristics are reported in Table S1. The vast majority of procedures were TEE guided (*n* = 92, 97%) with evidence of LAA thrombus reported at the beginning of the procedure in 6% of cases. All procedures were completed with successful implantation of Amulet (Abbott) (*n* = 39, 41%) in the majority of cases, followed by Watchman FLX (Boston Scientific) (*n* = 31, 33%) and Watchman 2.5 (*n* = 10, 11%). All procedures ended with no residual PDL. The main post‐LAAC antithrombotic therapy consisted of OAC alone (*n* = 54, 57%; of whom *n* = 41 under DOAC), followed by OAC in combination with single antiplatelet therapy (SAPT) (*n* = 24, 25%; of whom *n* = 20 under DOAC). Only in 16 (17%) cases did the post‐implantation therapy did not include any OAC: 15 patients were discharged under DAPT whereas only one under SAPT.

### 3.1 Study Endpoints and Analyses

**FIGURE 1:**
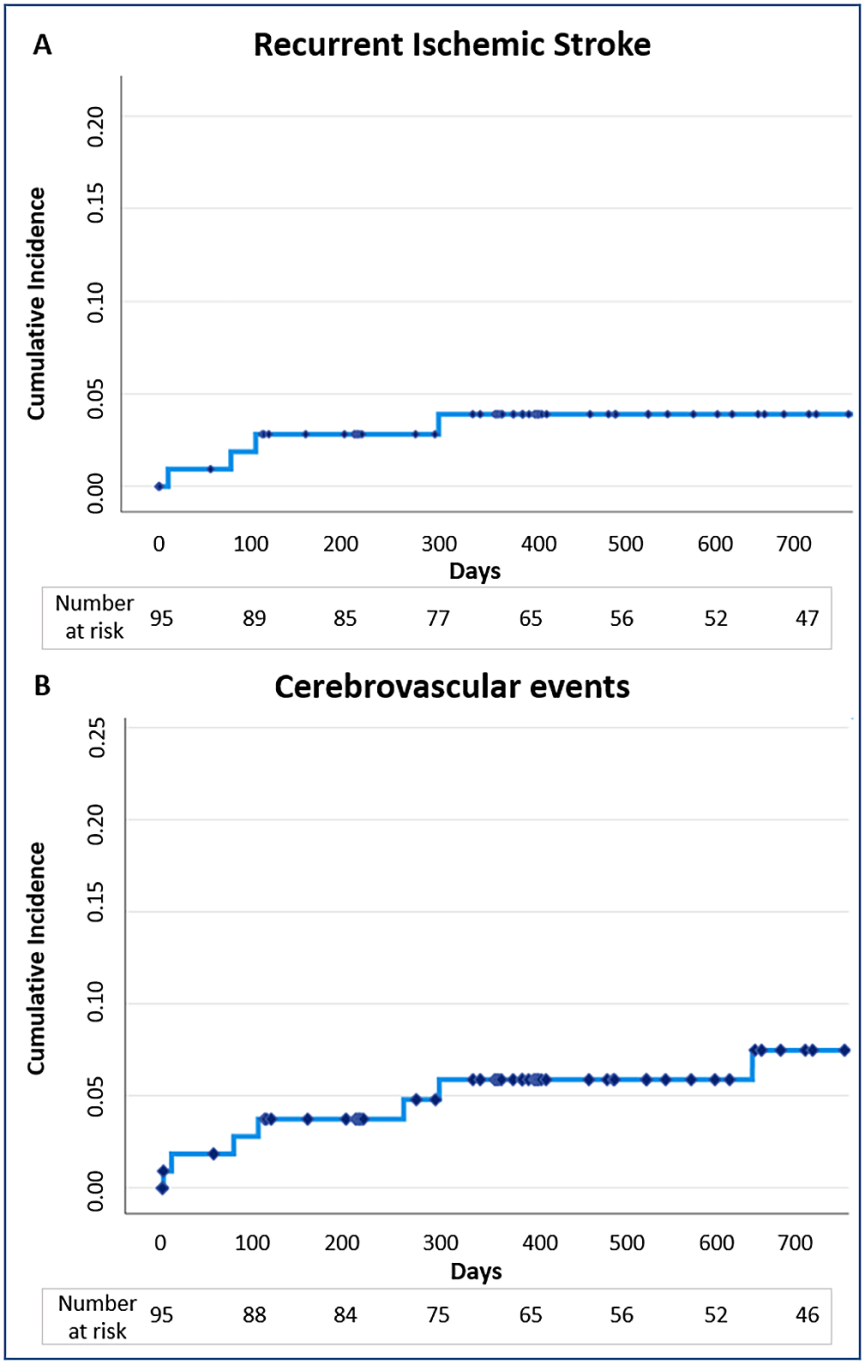
Cumulative incidence function curve of ischemic stroke (A) and cerebrovascular events (B) at 2 years after LAAC. Central illustration. Main study results. LAAC, left atrial appendage closure; OAC, oral anticoagulation; TIA, transient ischemic attack.

Clinical outcomes are shown in Table 2. At a median follow‐up of approximately 2 years (713 days [373–730]), when the majority of patients were still under OAC (*n* = 75, 83%; *n* = 61 under DOAC), the rate of recurrent ischemic strokes was 4% (*n* = 4). Details related to these 4 events are reported in Table 3. In 2 of them, the antithrombotic therapy at the recurrent stroke was DOAC: one event occurred in the context of an elective pulmonary vein isolation procedure performed 10 months after LAAC, whereas another event was categorized as related to small vessel disease. In the two remaining patients, where a cardioembolism event was suspected, the antithrombotic therapy consisted of VKA or DAPT. The mean National Institutes of Health Stroke Scale (NIHSS) of the 4 strokes was 5.5 ± 2.9. Almost 1 in 20 patients died (*n* = 5; 5%), whereas the rate of cerebrovascular events was 7% (*n* = 7). No haemorrhagic stroke was observed. The Kaplan–Meier curves relative to the observed ischemic stroke and thromboembolic events during follow‐up are reported in Figure 1. Procedure‐related complications were rare (*n* = 1; 1%) and included just one episode of TIA occurring 2 days after the intervention.

**TABLE 2:** Study endpoints and follow‐up.

	All patients (*N* = 95)
**2‐year clinical follow‐up**	
Clinical follow‐up duration (days), median (IQR)	713.0 (373.0; 730.0)
Ischemic stroke, *n* (%)	4 (4%)
Death, *n* (%)	5 (5%)
Cardiovascular death, *n* (%)	3 (3%)
Cerebrovascular events, *n* (%)	7 (7%)
Haemorrhagic stroke, *n* (%)	0 (0%)
Transient ischemic attack, *n* (%)	3 (3%)
BARC 3 or 5, *n* (%)	2 (2%)
BARC 3 or 5 non‐procedural, *n* (%)	2 (2%)
Procedural related complication (within 7 days after LAAC), *n* (%)	1 (1%)
**TEE follow‐up**	
TEE follow‐up performed, *n* (%)	91 (96%)
TEE follow‐up duration (days), median (IQR)	66.5 (51.3; 133.0)
Device related thrombus, *n* (%)	2 (2%)
Any peri‐device leak, *n* (%)	7 (9%)
Peri‐device leak (≥ 3 mm), *n* (%)	2 (2%)
**Antithrombotic therapy at follow‐up**	
OAC	67 (74%)
SAPT + OAC	5 (6%)
DAPT + OAC	3 (3%)
SAPT	9 (10%)
DAPT	5 (6%)
No antithrombotic therapy	1 (1%)

Abbreviations: BARC, Bleeding Academic Research Consortium; DAPT, dual antiplatelet therapy; IQR, interquartile range; LAAC, left atrial appendage closure; OAC, oral anticoagulant; SAPT, single antiplatelet therapy; TEE, transesophageal echocardiography.

**TABLE 3:** Patients with recurrent stroke during the follow‐up.

Patient	Number of days after LAAC	NIHSS	Antithrombotic therapy at discharge	Antithrombotic therapy at time of event	Device related complication at TEE follow‐up	Ischemic stroke etiology
1	79	7	VKA	VKA (INR 3.7)	No	Unclear (suspected cardioembolism)
2	11	9	DAPT	DAPT	No echocardiographic follow‐up^a^	Unclear (suspected cardioembolism)
3	106	1	ASA, DOAC (Dabigatran 150 mg)	DOAC (Dabigatran 150 mg)	No	Unclear (suspected small vessel disease due to severe microangiopathy)
4	304	5	ASA, DOAC (Apixaban 5 mg)	DOAC (Apixaban 5 mg)	No	Periprocedural after PVI procedure

Abbreviations: ASA, acetylsalicylic acid; DAPT, dual antiplatelet therapy; DOAC, direct oral anticoagulant; DRT, device‐related thrombus; INR, international normalized ratio; PDL, peridevice leak; PVI, pulmonary vein isolation; VKA, vitamin‐K antagonist.

^a^ Patient died before echocardiographic follow‐up due to hemorrhagic transformation of the stroke.

A TEE follow‐up was performed in the vast majority of patients (96%, *n* = 91) at 2 months after the procedure. DRT and any residual PDL were reported in 2% (*n* = 2) and 9% (*n* = 7) of patients, respectively.

Cerebrovascular events (6% vs. 13%; *p* = 0.340) were numerically, albeit non‐significantly, lower in patients discharged with or without OAC and were similar between patients receiving a double or a single closure system device (8.1% vs. 10.8%; *p* = 0.718).

The observed thromboembolic event rate at 1 year was 6% (95% CI: 1%–11%), which translates into a 21% risk reduction (Standardized Event Ratio: 0.79; 95% CI: 0.36–1.77) compared to the 7.6% predicted by baseline CHA_2_DS_2_‐VASc score (5.4) for AF patients without OAC.

## 4 Discussion

Up to 40% of strokes in AF patients occur despite OAC [24]. The management of these patients is challenging since, despite the high risk of stroke recurrence, the optimal prevention strategy has not yet been determined [3, 24]. LAAC is a promising strategy, but data supporting this option are still limited, especially when considering the patient's selection modality and the optimal procedure timing.

In light of this evidence gap limiting the generalizability of the so far available data, we have conducted this multicenter and multidisciplinary analysis, which main findings can be summarized as follows (Graphical Abstract):

1. Approximately 4 months after a cardioembolic breakthrough stroke, the LAAC procedure showed excellent results in terms of technical success and device‐ or procedure‐related complications.
2. The combination of LAAC and OAC after a cardioembolic breakthrough stroke was associated with a numerically lower risk of stroke recurrence compared to previous breakthrough stroke studies with conservative management and to the expected stroke rate.

### 4.1 LAAC Timing

Patients who experience a stroke while on OAC represent a subgroup of AF patients at exceptionally high risk for recurrent ischemic events, especially during the first few months after the event. A recent multinational study, which included 2946 AF patients with breakthrough stroke between January 2012 and December 2020, showed that approximately one in four patients experienced an adverse event—such as death, ischemic stroke, or intracranial bleeding—within 3 months after the index stroke [5]. The ideal timing for performing the LAAC procedure in such patients is still unknown due to lacking data (Table 4). In our study, patients underwent the LAAC procedure approximately 4 months after the index stroke, with excellent procedural outcomes: 100% technical success, only one procedure‐related complication (one TIA), and low rates of DRT and PDL at TEE follow‐up. These promising procedural outcomes align with those reported in similar studies (Table 4), confirming that the LAAC procedure appears feasible and safe in this high‐risk population [12, 13, 25, 26, 27, 28]. However, it remains unclear whether performing the LAAC procedure earlier than 4 months after the index stroke could mitigate early‐occurring events or potentially increase procedural risks. Future comparative studies between early and late approaches are needed to enhance our understanding of this aspect.

**TABLE 4:** Observational studies including patients with AF and ischemic stroke despite OAC submitted to LAAC^a^.

	Study design	No. of patients	Age (mean)	CHA^2^DS^2^‐VASc score (mean)	Index stroke definition	Index stroke adjudication by neurologist	Delay between index stroke and LAAC (days)	Device used	Main antithrombotic therapy at discharge	Ischemic stroke mean rate per 100 patient years	Mean follow‐up duration (years)
Cruz et al. [13] 2020	MC‐RS	115	73.8	5.5	Previous stroke on OAC	No	NA	ACP	NA	1.9	1.4
Pracon et al. [25] 2022	SC‐PS	39	73*	5^b^	Ischemic stroke, TIA, SE or LAA Thrombus despite OAC	No	NA	Amulet, Watchman 2.5	DAPT (100% of cases)	7.7^c^	1.0
Margonato et al. [26] 2022	SC‐RS	102	69	3^b^	Thromboembolic event or sludge in the LAA despite OAC	No	NA	Amulet, Watchman 2.5 Watchman FLX	OAC alone (70% of cases)	1.9^c^	3.9^b^
Aarnink et al. [27] 2024	MC‐RS	438	72.0	5.0	Ischemic stroke, TIA, SE or LAA Thrombus despite OAC	No	NA	Watchman 2.5, Watchman FLX, ACP, Amulet	OA alone (54.3% of cases)	2.5	2.2
Fouks et al. [12] 2024	MC‐RS	29	73.4	6.0	Ischemic stroke despite adequate OAC	Yes	NA	Watchman 2.5, Watchman FLX	OAC alone (93.1% of the cases)	2.0	1.8
Maarse et al. [28] 2024	MC‐RS	433	71.8	5.0	Ischemic stroke, TIA, SE or LAA Thrombus despite OAC	Yes	NA	Watchman 2.5, Watchman FLX, ACP, Amulet, LAmbre, Coherex Wavecrest	OAC alone (60.0% of cases)	2.8	2.0

Abbreviations: AF, atrial fibrillation; ACP, Amplatzer cardiac plug; ASA, acetylsalicylic acid; CE, cardioembolic; LAA, left atrial appendage closure; MC, multicenter; NA, not available; OAC, oral anticoagulant; PS, prospective; RS, retrospective; SC, single‐center; SE, systemic embolism; TIA, transient ischemic attack.

^a^ Only studies including at least 25 patients have been included.

^b^ Median instead of mean.

^c^ Cumulative rate (%) at the relative follow‐up time. refers to median.

### 4.2 Ischemic Stroke Recurrence

At a median follow‐up of 2 years after LAAC, we observed a recurrence rate of ischemic strokes of 4%. This result is consistent with the mean ischemic stroke rates (1.9–2.8 per 100 patient‐years) reported in previous similar LAAC studies (Table 4) and lower compared to the rates reported in breakthrough stroke cohorts without LAAC (5.3–8.9 per 100 patient‐years) [29, 30, 31, 32]. These observations are promising and support the hypothesis that LAAC, in combination with OAC, might be used as a potential strategy to further reduce the stroke recurrence risk in this high‐risk population. However, the comparison of outcomes between different cohort studies is very limited, especially considering two aspects. First, the follow‐up for stroke recurrence in studies including breakthrough stroke patients treated with LAAC + OAC (including ours) generally starts after the LAAC procedure, whereas in studies involving breakthrough stroke patients treated with OAC, the follow‐up begins from the index event, when the recurrence risk is potentially the highest. Second, LAAC studies might have included a selected subgroup of AF patients with breakthrough stroke, characterized by a lower risk profile (since they survived the first 3 months after the stroke and were considered eligible for the interventional procedure). Similar limitations also affected the recently published propensity score–matching analysis comparing two multicenter cohorts of AF patients who experienced a cerebrovascular event despite OAC and then either underwent LAAC (STR‐OAC LAAO cohort) or continued OAC (control cohort) [28]. The authors showed that, during the 2‐year follow‐up, the annualized event rates for ischemic stroke were 2.8% for the STR‐OAC LAAO cohort and 8.9% for the control cohort [28]. Furthermore, the authors observed a significantly different time to the first ischemic stroke between the two cohorts (HR, 0.33; 95% CI: 0.19–0.58; *p* < 0.001) [28].

### 4.3 Breakthrough Stroke as Indication to LAAC

LAAC procedure is an established therapeutic option for preventing stroke in patients with AF and has been assigned a Class 2a (in the United States) and Class 2b (in Europe), Level of Evidence “B” indication for patients with a contraindication to long‐term OAC due to a nonreversible cause [6, 33]. As a result, the majority of LAAC procedures are now performed in patients deemed ineligible for long‐term OAC. In our multicenter cohort, stroke occurrence despite OAC was the clinical indication for LAAC in only 5% of patients treated at the participating centers, a percentage similar to the 2.3% reported by the largest randomized clinical trial on LAAC to date [34]. The low percentages related to this indication are indeed due to the limited level of evidence still supporting this procedure.

### 4.4 Future Perspectives

Two RCTs are currently testing the impact of percutaneous LAAC in further reducing ischemic stroke in AF patients on OAC. ELAPSE (NCT05976685) is an investigator‐initiated international superiority RCT, which will use an adaptive design including minimally 482 and maximally 1000 patients with AF and cardioembolic breakthrough stroke. It compares the continuation of OAC alone with the combination of LAAC plus OAC within 3 months of the index stroke, in terms of a composite of cardiovascular death, stroke, or systemic embolism [35]. The LAAOS‐4 trial (NCT05963698) is a sponsored international superiority RCT that includes approximately 4000 patients with AF and a CHA_2_DS_2_‐VASc score of ≥ 4 but not necessarily a history of breakthrough stroke. It compares OAC alone with the combination of LAAC plus OAC in terms of a composite of ischemic stroke or systemic embolism. Both trials have just begun enrollment, and results are not expected before 2028.

### 4.5 Limitations

The present analysis has certain limitations that should be acknowledged. First, the retrospective design may have introduced bias and unmeasured confounders. Second, no control group was available. Third, both the index stroke etiologies and the cerebrovascular events observed during follow‐up were not centrally adjudicated but were instead locally adjudicated by stroke neurologists. Fourth, events that occurred between the index stroke and the LAAC procedure were not collected. Fifth, the small sample size reduces the generalizability of the study findings. Sixth, OAC was not maintained long‐term after LAAC in approximately 15% of patients. Finally, the three subanalyses are restricted to secondary outcomes due to the low event rate for the primary endpoint.

## 5 Conclusions

In this European cohort of consecutive AF patients who underwent LAAC due to cardioembolic stroke despite optimal OAC, LAAC performed 4 months after the index stroke appeared feasible and safe. The 2‐year stroke recurrence rate suggests good efficacy when combining LAAC and OAC in preventing stroke in this high‐risk population. Results from ongoing trials comparing the combination of LAAC plus OAC with OAC alone are needed.

## Ethics Statement

The study received approval from the local ethical committee at each participating site.

## Consent

Written informed consent was obtained from all subjects before the study.

## Conflicts of Interest

R.G. is a proctor and consultant for Abbott and reports speaking honorarium from Boston Scientific and research grants from Swiss Heart Foundation. E.A. reports research funding from the Gottfried & Julia Bangerter Rhyner foundation. U.F. reports research grants from the Swiss National Science Foundation, the Swiss Heart Foundation, from Medtronic (BEYOND SWIFT, SWIFT DIRECT) and from Stryker, Rapid medical, Penumbra, Medtronic and Phenox (DISTAL), Boehringer Ingelheim (TECNO). Support of the Horton Foundation for the DO IT trial. Consultancies for Medtronic (fees paid to institution). Participation in an advisory board for AstraZeneca (former Alexion/Portola), Bayer, Boehringer Ingelheim, Biogen, AbbVie, Siemens (fees paid to institution). Member of a clinical event committee (CEC) of the COATING study (Phenox). Member of the data and safety monitoring committee (DSMB) of the TITAN, LATE_MT, IN EXTREMIS and RapidPulse trials. L.R. reports research grants to institution by Abbott‐Vascular, Boston‐Scientific, Biotronik, Heartflow, Sanofi, Regeneron. He reports speaker/consultation fees by Abbott‐Vascular, Amgen, AstraZeneca, CSL‐Behring, Canon, Occlutech, Sanofi, Vifor. All other authors have reported that they have no relationships relevant to the contents of this paper to disclose.

## Supporting information


**Data S1:** ene70365‐sup‐0001‐DataS1.docx.

**Data S2:** Reporting checklist for cohort study.

## Data Availability

The data that support the findings of this study are available from the corresponding author upon reasonable request.
