# Supplementary material for: Percutaneous Left Atrial Appendage Closure in Patients With Cardioembolic Breakthrough Stroke: An International Observational Study
Source: Eur J Neurol. 2025 Sep 23;32(9):e70365. doi: 10.1111/ene.70365 (PMC12455140; doi:10.1111/ene.70365)
Supplement: Supplementary file 1 — Data S1: ene70365‐sup‐0001‐DataS1.docx. [file ENE-32-e70365-s002.docx]

SUPPLEMENTAL MATERIAL

**Percutaneous Left Atrial Appendage Closure in Patients with Cardioembolic Breakthrough Stroke: an International Observational Study**

Galea et al.

TABLE OF CONTENTS

**Supplemental Figure I** - Patient flow chart……………..…..……………………………………………2

**Supplemental Table I** - Procedural Characteristics ………………………………………….…………..3

**Supplemental Table II-** Bleeding Classification………………………………………………………….5

**Supplemental Figure I** - Patient flow chart

**
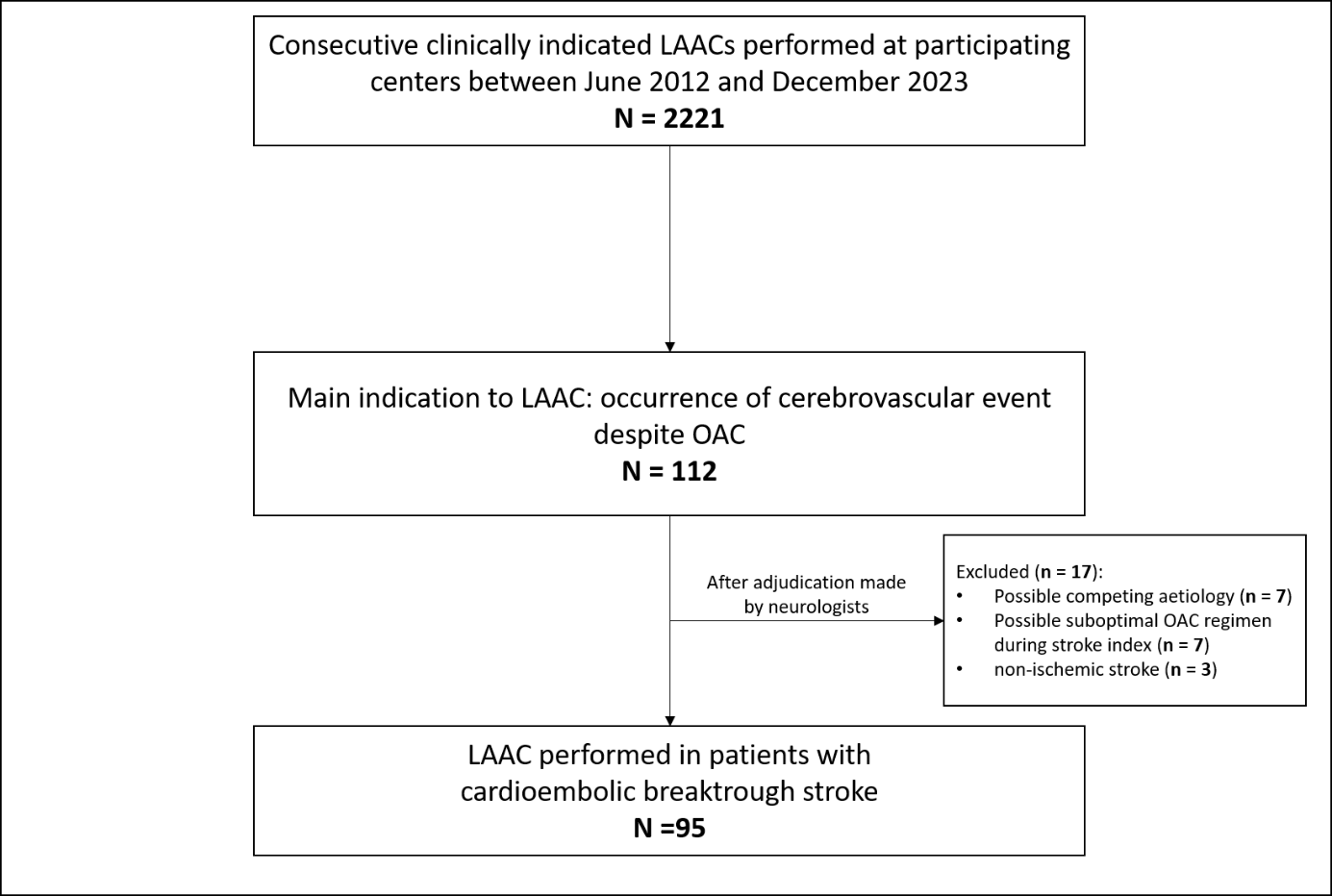
**

LAAC, Left Atrial Appendage Closure; OAC, Oral Anticoagulation.

**Supplemental Table I** - Procedural Characteristics

|  | All Patients (N =95) |
| --- | --- |
| TEE guidance, n (%) | 92 (97%) |
| LAA thrombus at pre/intraprocedural TEE, n (%) | 6 (6%) |
| General anesthesia, n (%) | N=28; 5 (18%) |
| Sinus rhythm during procedure, n (%) | N=26; 10 (38%) |
| Time of procedure (min), mean ± SD | N=81; 57.4 ± 28.3 |
| Technical Success, n (%) | 95 (100%) |
| LAAC Device implanted |  |
| Watchman 2.5, n (%) | 10 (11%) |
| Watchman FLX, n (%) | 31 (33%) |
| Amulet, n (%) | 39 (41%) |
| Lambre, n (%) | 13 (13%) |
| Omega, n (%) | 1 (1%) |
| ACP, n (%) | 1 (1%) |
| Discharge antithrombotic therapy |  |
| OAC | 54 (57%) |
| SAPT+OAC | 24 (25%) |
| DAPT+OAC | 1 (1%) |
| SAPT | 1 (1%) |
| DAPT | 15 (16%) |

ACP, Amplatzer cardiac plug; DAPT, Dual antiplatelet therapy; LAA, Left atrial appendage; OAC, oral anticoagulant; SAPT, Single antiplatelet therapy; SD, Standard Deviation; TEE, Transesophageal Echocardiography

**Definitions of study endpoints**

1. **STROKE**

Stroke was defined as brain, spinal cord, or retinal cell death attributable to ischemia or hemorrhage, based on at least one of the following conditions:

-clinical evidence of focal ischemic injury with symptoms persisting ≥24 hours or until death and without any possible etiologies, OR

-objective evidence of focal ischemic injury in a defined vascular distribution.^6^

Based on this definition, stroke endpoint were adjudicated taking into account three elements: the neurological dysfunction, absence of a nonvascular mechanism, and imaging evidence of ischemic injury. The neurologic dysfunction consisted of an acute episode of a focal or global neurological deficit with at least one of the following: change in the level of consciousness, hemiplegia, hemiparesis, one-sided numbness or sensory loss, dysphasia or aphasia, hemianopia, amaurosis fugax, any other neurological signs or symptoms consistent with stroke. Furthermore, duration of neurological dysfunction longer than 24h helped to exclude transient ischemic attack (TIA). Other readily identifiable non-stroke cause for the clinical presentation (e.g. brain tumour, trauma, infection, hypoglycaemia, peripheral lesion, pharmacologic influences) were excluded based on clinical/imaging assessment. The objective evidence of focal ischemic injury were performed by means of neuroimaging procedure (computed tomography (CT) scan or brain magnetic resonance imaging (MRI)) findings. Again, imaging-documented new haemorrhage or infarction were useful to exclude TIA in the event that duration of neurological dysfunction was shorter than 24 h.

Classification:

**Ischemic Stroke**

Ischemic stroke was defined as an acute episode of focal cerebral, spinal, or retinal dysfunction caused by CNS infarction. Evidence of infarction was defined as pathological, imaging, or other objective evidence of acute cerebral, spinal cord, or retinal focal ischemic injury in a defined vascular distribution. In absence of the above (i.e. imaging or autopsy unavailable), clinical evidence of cerebral, spinal cord, or retinal focal ischemic injury was based on symptoms persisting ≥24 hours or until death, after excluding other etiologies. Note, hemorrhagic infarction, defined as a parenchymal hemorrhage after CNS infarction, was considered an ischemic stroke.

- **Haemorrhagic Stroke**

Haemorrhagic Stroke was defined as a rapidly developing clinical signs of neurological dysfunction (focal or global) attributable to a focal collection of blood within the brain parenchyma, ventricular system or subarachnoid space (the space between the arachnoid membrane and the pia mater of the brain or spinal cord), that was not caused by trauma. Hemorrhages in the CNS were classified as stroke if they were nontraumatic, caused by a vascular event, and resulted in injury to the CNS. In contrast, traumatic hemorrhages and subdural hematoma were not characterized as stroke. The diagnoses included in this section were intracerebral hemorrhage (intraparenchymal and intraventricular) and subarachnoid hemorrhage (both aneurysmal and nonaneurysmal).

- **Undetermined Stroke**

Undetermined Stroke was defined as an acute episode of focal or global neurological dysfunction persisting ≥24 hours (or until death) caused by presumed brain, spinal cord, or retinal vascular injury as a result of haemorrhage or infarction but with insufficient information to allow categorization as an ischaemic or haemorrhagic stroke.

1. **PROCEDURE RELATED COMPLICATIONS**

Procedure related complications were defined as adverse clinical events occurring within 7 days after procedure. In particular, the following complications were adjudicated: death, TIA/stroke, systemic embolism or major bleedings.

1. **SYSTEMIC EMBOLIM**

Systemic embolism was defined as an acute vascular insufficiency or occlusion of the extremities or any non-CNS organ associated with clinical, imaging, surgical/autopsy evidence of arterial occlusion in the absence of other likely mechanism (e.g. trauma, atherosclerosis, or instrumentation). When there was presence of prior peripheral artery disease, angiographic or surgical or autopsy evidence was required to show abrupt arterial occlusion.

1. **BLEEDINGS**

All potential bleeding events were adjudicated according to Bleeding Academic Research Consortium (BARC) classification (Supplemental Table 2).

**Supplemental Table 2**

| Type 0 | No bleeding |
| --- | --- |
| Type 1 | Bleeding that is not actionable and does not cause the patient to seek unscheduled performance of studies, hospitalization, or treatment by a health care professional. May include episodes leading to self-discontinuation of medical therapy by the patient, without consulting a health care professional. |
| Type 2 | Any overt, actionable sign of hemorrhage (e.g. more bleeding than would be expected for a clinical circumstance; including bleeding found by imaging alone) that does not fit the criteria for Types 3, 4, or 5 but does meet at least one of the following criteria:  -Requiring non-surgical, medical intervention by a health care professional  -Leading to hospitalization of increased level of care  -Prompting evaluation |
| Type 3a  Type 3b  Type 3c | Overt bleeding plus hemoglobin drop of 3 to <5* g/dL (provided hemoglobin drop is related to bleed)  Any transfusion with overt bleeding  Overt bleeding plus hemoglobin drop ≥5* g/dL (provided hemoglobin drop is related to bleed)  Cardiac tamponade  Bleeding requiring surgical intervention for control (excluding dental / nasal / skin / hemorrhoid)  Bleeding requiring intravenous vasoactive agents  Intracranial hemorrhage (does not include microbleeds or hemorrhagic transformation; does include intraspinal)  Subcategories: confirmed by autopsy or imaging or lumbar puncture  Intra-ocular bleed compromising vision |
| Type 4 | CABG-related bleeding  Perioperative intracranial bleeding within 48 hours  Reoperation following closure of sternotomy for the purpose of controlling bleeding Transfusion of ≥ 5 units of whole blood or packed red blood cells within 48 hour period†  Chest tube output ≥ 2 L within a 24 hour period |
| Type 5a  Type 5b | Probable fatal bleeding; no autopsy or imaging confirmation, but clinically suspicious  Definite fatal bleeding: overt bleeding or autopsy or imaging confirmation |

* Corrected for transfusion (1 U packed red blood cells or 1 U whole blood_1g/dL hemoglobin). † Cell saver products will not be counted.
